# Effect of climate change on *Aspergillus flavus* and aflatoxin B_1_ production

**DOI:** 10.3389/fmicb.2014.00348

**Published:** 2014-07-22

**Authors:** Angel Medina, Alicia Rodriguez, Naresh Magan

**Affiliations:** Applied Mycology Group, Cranfield Soil and AgriFood Institute, Cranfield UniversityCranfield, Bedford, UK

**Keywords:** climate change factors, water activity, temperature, elevated CO_2_, growth, gene expression, aflatoxin production, ecology

## Abstract

This review considers the available information on the potential impact of key environmental factors and their interactions on the molecular ecology, growth and aflatoxin production by *Aspergillus flavus in vitro* and in maize grain. The recent studies which have been carried out to examine the impact of water activity × temperature on aflatoxin biosynthesis and phenotypic aflatoxin production are examined. These have shown that there is a direct relationship between the relative expression of key regulatory and structural genes under different environmental conditions which correlate directly with aflatoxin B1 production. A model has been developed to integrate the relative expression of 10 biosynthetic genes in the pathway, growth and aflatoxin B_1_ (AFB_1_) production which was validated under elevated temperature and water stress conditions. The effect of interacting conditions of a_w_ × temperature × elevated CO_2_ (2 × and 3 × existing levels) are detailed for the first time. This suggests that while such interacting environmental conditions have little effect on growth they do have a significant impact on aflatoxin biosynthetic gene expression (structural *aflD* and regulatory *aflR* genes) and can significantly stimulate the production of AFB_1_. While the individual factors alone have an impact, it is the combined effect of these three abiotic factors which have an impact on mycotoxin production. This approach provides data which is necessary to help predict the real impacts of climate change on mycotoxigenic fungi.

## Introduction

Food security has become a very important issue world-wide and the potential effects of climate change on yields and quality of food is now receiving significant attention by scientists, especially from a risk analysis perspective. The moldy contamination of staple foods such as cereals has received attention because of their acute and chronic effects in humans and animals. Indeed, the increasing use of staple crops, especially maize for biofuel production, has put further pressure on such key food crops. There is particular interest in maize because it is a key staple food in both developed and developing regions world-wide. Maize is prone to infection by *Aspergillus flavus* and *Aspergillus parasiticus*, especially via insect damage during silking and contamination with aflatoxins. Aflatoxins have been rated as class 1A carcinogens by the International Agency for Research of Cancer (IARC, 2012). They are heat stable and difficult to destroy during processing. Thus exposure, both acute and chronic, can have significant impacts on vulnerable groups, especially babies and children. This has resulted in strict legislative limits in many parts of the world for aflatoxins and mycotoxins in a wide range of foodstuffs (European Commission., 2006). However, in African countries where legislation is often applied to export crops only, consumption of mycotoxin contaminated staple foods is a significant risk, with rural populations exposed to aflatoxins throughout their lives, with serious impacts on their health (Wagacha and Muthomi, 2008). This is exemplified by the relatively recent acute outbreak of severe aflatoxicosis in Kenya (Lewis et al., 2005).

Climate change is expected to have a profound effect on our landscape world-wide. For some areas, climatic models have projected a marked decrease in summer precipitation and increases in temperature, which would result in concomitant drought stress episodes. The environment in which crops will be grown in the next 10–25 years may change markedly with atmospheric CO_2_ concentrations expected to double or triple (from 350 to 700 or 900–1000 ppm). Thus, there has been a lot of interest in the impact that climate change scenarios may have on economically important crops/mycotoxigenic fungal infection and contamination with mycotoxins (Paterson and Lima, 2010, 2011; Magan et al., 2011; Wu et al., 2011). Indeed, climate change conditions may impact on the interactions between different mycotoxigenic species and indeed other mycobiota and determine the relative mycotoxin composition contaminating staple foods/feeds (Magan et al., 2010; Paterson and Lima, 2012). Because of this increase and that of other greenhouse gases, the global temperature is expected to increase by between +2 and +5°C.

The EU green paper on climate change in Europe also suggests that effects will be regional and be either detrimental or advantageous depending on geographical area. Thus, in Southern Europe, changes may equate to an increase of 4–5°C with longer drought periods, resulting in increasing desertification, and a decrease in crop yields. In areas of Western and Atlantic Europe, changes of 2.5–3.5°C with dryer and hotter summers are envisaged. In Central Europe, an increase of 3–4°C, higher rainfall and floods are forecast, although longer growing periods may benefit crop yields. Northern Europe would expect a mean temperature increase of 3–4.5°C, with a significant increase in precipitation of 30–40%. This may lead to increases in crop yields and perhaps new crop cultivation patterns (European Commission., 2007; Solomon et al., 2007). Similar impacts have been described in other areas of the world, especially parts of Asia and Central and South America which are important producers of staple crops (IPCC, 2007). A recent study has predicted that, on a global scale, pests, and diseases are moving to the poles at the rate of 3–5 km/year (Bebber et al., 2013). This could have further impacts on contamination of staple foods such as maize, as increases in pest reproduction rates will lead to more damage and facilitate more infection by *A. flavus* and contamination with aflatoxins. However, in the recent predictions by Bebber et al. (2013) no focus on spread of mycotoxigenic fungi or mycotoxins or interactions between pathogens and pests were considered in the context of climate change.

In developing countries drought stress may be particularly important in terms of food security. For example, marginal land where stress tolerant sorghum was previously grown has now been replaced with maize in both West and East Africa. Maize as well as ground nuts are particularly prone to infection when water stress periods occur. This leads to increased aflatoxin contamination of such crops pre-harvest and post-harvest and can significantly impact on the ability to export the crop and also on the nutritional quality when consumed in rural subsistence communities.

Magan et al. (2011) suggested that climate change factors may result in xerophilic fungi such as *Wallemia sebi*, *Xeromyces bisporus*, and *Chrysosporium* species becoming more important as colonizers of food commodities, as they can grow under very dry conditions [0.65–0.75 water activity (a_w_)] where there is much less competition from the majority of mesophilic fungi (Magan, 2006; Magan and Aldred, 2007). For example, *W. sebi* can produce metabolites such as walleminol and walleminone which can be toxic to animals and humans (Piecková and Kunová, 2002). Studies also suggest that there are competitive interactions between these xerophilic fungi in dry and hot conditions and that secondary metabolites may play a role (Leong et al., 2010). This will certainly have an impact on agricultural productivity, especially of essential/staple food crops such as maize and nuts and also influence the interface between plants, insect pests and fungal infection of staple foods (Miraglia et al., 2009). This could have a profound effect on pre- and post-harvest mycotoxin contamination, especially aflatoxins in developing countries, where food quality and security issues are critical.

Examples of modified weather regimes impacting on mycotoxins were demonstrated by the 2003/2004 and subsequently in 2012 summer seasons in the Mediterranean region such as Northern Italy where drought and elevated temperatures resulted in a switch from *Fusarium verticillioides* and contamination with fumonisins to significant contamination of maize grain with *A. flavus* and aflatoxins and entry of aflatoxin M_1_ into the dairy chain via the animal feed chain (Giorni et al., 2007). More recently, a survey of Serbian maize samples in 2009–2011 contained no aflatoxins. However, prolonged hot and dry weather in 2012 resulted in 69% of samples containing aflatoxins (Kos et al., 2013). Similarly in Hungary it has also been shown that an increase in aflatoxins may be due to climate change conditions (Dobolyi et al., 2013). However, previous to these examples there are only a few concrete examples of such incidences where climate change factors have been implicated (Magan et al., 2011).

## Effect of water stress × temperature stress effects on aflatoxin cluster gene expression, growth, and aflatoxin production

**Figure 1:**
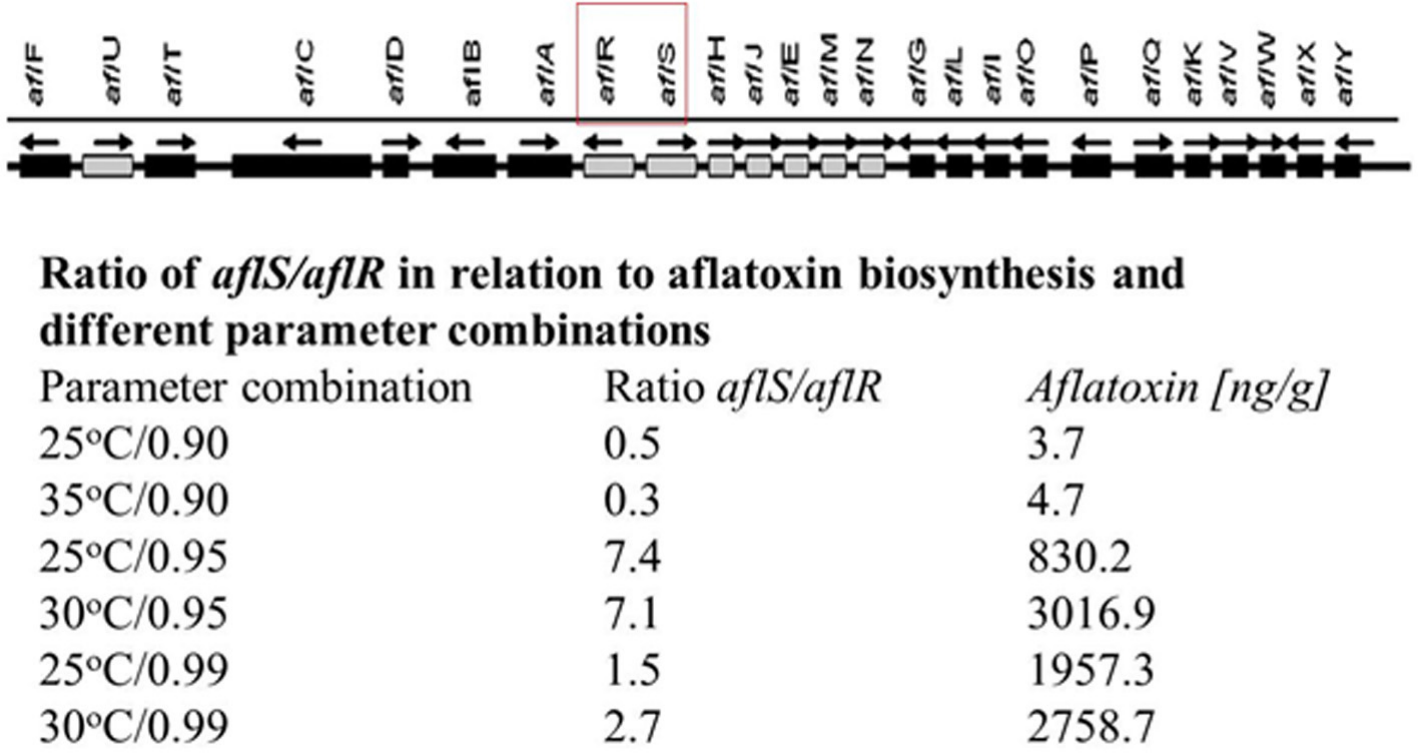
**The key structural and regulatory genes involved in aflatoxin production and the effect of temperature × water activity conditions, ratio of the two regulatory genes and relative amounts of aflatoxin B_1_ production (adapted from Schmidt-Heydt et al., 2010)**.

Generally, the aflatoxin biosynthesis genes of *A. flavus* and *A. parasiticus* are highly homologous and the order of the genes (approx. 30) within the cluster has been shown to be the same (Yu et al., 1995, 2004). These include key regulatory genes (*aflR* and *aflS*) and a series of up and downstream structural genes. It has been shown that both water availability and temperature modifications affect the expression of these clusters of genes, relative growth rate and aflatoxin production in both *A. flavus* and *A. parasiticus* (Schmidt-Heydt et al., 2010, 2011). It was shown that there was a good correlation between the expression of an early structural gene (*aflD*) and aflatoxin B_1_ (AFB_1_) (Abdel-Hadi et al., 2010). It has also been shown that temperature × a_w_ interactions were related to the ratio of the two key regulatory genes (*aflR/aflS*). The higher the ratio, the higher the relative AFB_1_ production (Schmidt-Heydt et al., 2009, 2010; Figure 1). This suggests that under certain interacting conditions of two environmental stress factors significantly influences on the relative amounts of AFB_1_ produced.

**Figure 2:**
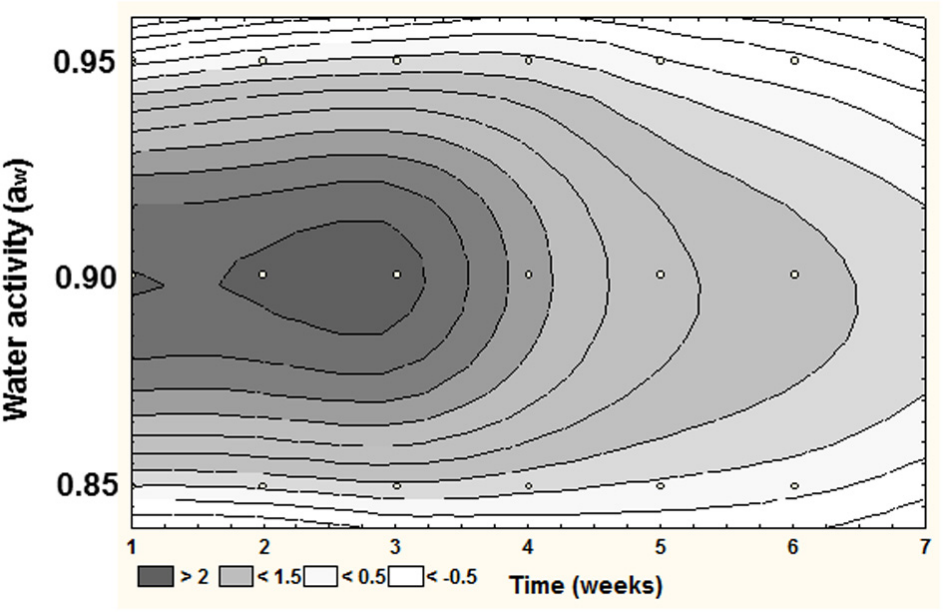
**Relative expression of *aflD (nor1)* gene during colonization of peanuts by *Aspergillus flavus* during storage**. Optimum expression occurred at 0.90 a_w_ during the first 2–3 weeks of storage at 25°C (from Abdel-Hadi et al., 2010).

The study by Abdel-Hadi et al. (2010) also showed that when examining the relationship between temporal AFB_1_ production and the relative expression of the *aflD* structural gene involved early in the biosynthetic pathway then the relative expression could be mapped over time (Figure 2). This also suggests that the optimum a_w_ for *aflD* expression was at 0.90 a_w_, which is different from that for growth (0.95 a_w_).

**Figure 3:**
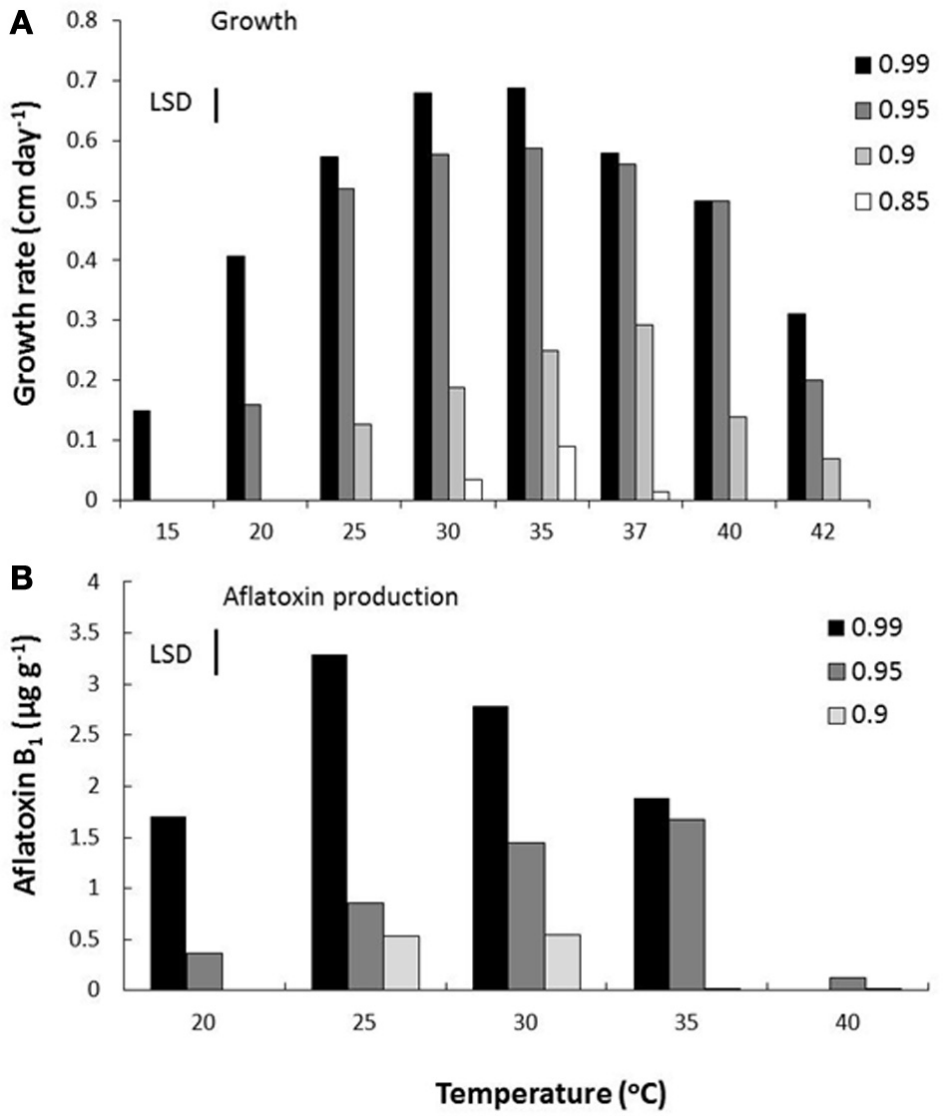
**Effect of water activity and temperature on **(A)** growth and **(B)** aflatoxin B_1_, production by a strain of *A. flavus* (Abdel-Hadi et al., 2012)**. Bars indicate least Significant Differences.

**Figure 4:**
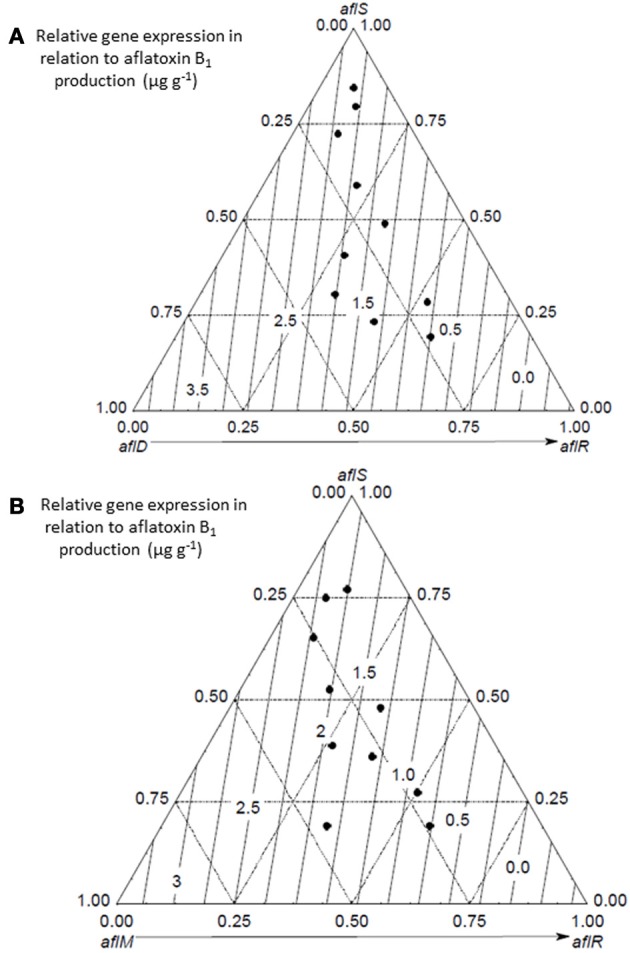
**Ternary diagrams of the relationship between the relative expression of the two regulatory genes *(aflS,aflR)* structural genes *(aflD,aflM)* on aflatoxin B_1_ production (μg g^−1^) (from Abdel-Hadi et al., 2012)**.

More recent detailed studies using a mycotoxin microarray (Schmidt-Heydt and Geisen, 2007) have been useful in elucidating the relationship between both key structural genes and the regulatory genes and interacting conditions of a_w_ × temperature and integrated the data on relative expression of 10 genes with growth and AFB_1_ data (Abdel-Hadi et al., 2012). Figure 3 shows the effect of these factors on growth and AFB_1_ production. They were able to model and validate this relationship under elevated temperature and drought stress conditions but elevated CO_2_ was not included in these studies. However, the relative relationship between the regulatory genes and key structural genes were examined using relative expression data under conditions of changing temperature and water stress to better understand the relationships between the regulatory and structural genes (Figure 4). This development of such ternary diagrams can help to evaluate the relationships between 3 key regulatory and structural genes at a time under different temperature and water stress conditions to help identify which are critical in the biosynthetic pathway as environmental factors change.

Schmidt-Heydt et al. (2008) also demonstrated that there was a stimulation of toxin biosynthetic gene expression in different mycotoxigenic fungi when exposed to interacting a_w_ × temperature stresses including *A. flavus*. They suggested that there was two peaks of expression one under optimum abiotic interacting conditions and one when water and temperature stress was applied. Studies by Yu et al. (2011) examined the effect of elevated temperature on the relative expression of the whole genome of a type strain of *A. flavus* to identify groups of up and down regulated genes. However, these studies were carried out over short time periods and did not include interactions with environmental stresses or with elevated CO_2_.

A significant amount of data exists on the effect of interactions between water availability and temperature on the life cycle of mycotoxigenic fungi and mycotoxin production (Sanchis and Magan, 2004; Magan and Aldred, 2007). This includes the ecological conditions of a_w_ × temperature which will facilitate growth and AFB_1_ production. The a_w_ × temperature boundary conditions for toxin production are slightly different from that for growth. Based on this information it is possible to predict the effect of increased temperature (e.g., 37°C) and water stress (0.95, 0.90 a_w_) on growth and aflatoxin production (Table 1). This shows that as you increase the temperature to 37°C you get significantly less AFB_1_ produced although the *A. flavus* is able to grow. However, this excludes the interaction with CO_2_ which is necessary to examine in more detail the impact of predicted climate change scenarios.

**Table 1:** **Changes in growth and toxin production by *Aspergillus flavus* due to increase in temperature by +3 and +5°C at different water stress conditions**.

**Growth**				**Aflatoxin B_1_ production**			
**a_w_**	**μ max range/T**	**μ+3**	**μ+5**	**a_w_**	**τ max range/T**	**τ +3**	**τ +5**
0.95	6.9/35	5.6	5.0	0.95	3082–2278/37	102–138	6.1-NP
0.90	2.9/37	1.4	0.7	0.90	448.5–331.5/37	1-NP	NP

*Key: μ max, Maximum growth rate (mm day^−1^); μ+3, Growth rate increasing 3°C; μ+5, Growth rate increasing 5°C; τ max, Maximum toxin production (μg g^−1^); τ+3°C, Predicted toxin increasing +3°C; τ+5°C, Predicted toxin increasing +5°C, NP, No toxin production*.

## Climate change impacts (a_w_ × temperature × CO_2_) on aflatoxin gene cluster expression, growth and toxin production

Recently, Medina et al. (in press) examined the effect of existing environmental conditions and when conditions were changed from 34 to 37°C, with drought stress and CO_2_ was increased to 650 and 1000 ppm. They examined the effects on growth of *A. flavus* and on the relative expression of the structural *aflD* and the regulatory *aflR* genes, as well as AFB_1_ production for the first time. These studies have shown that for growth of *A. flavus* there was relatively little effect of these interacting climate change conditions (Table 2). However, there were significant differences between growth in relation to a_w_, but no effect of a_w_ × temperature × CO_2_ on growth rate.

**Table 2:** **Comparison of growth of *Aspergillus flavus* under different interacting conditions of elevated temperature, drought stress, and elevated CO_2_*in vitro* on a conductive yeast-glucose medium (adapted from Medina et al., in press)**.

		**Diametric growth (mm/day)**					
**Temperature**		**34°C**			**37°C**		
Water activity		0.97	0.95	0.92	0.97	0.95	0.92
Carbon dioxide (ppm)	350	12.4	11.7	6.8	10.2	9.8	7.3
	650	12.1	11.6	6.9	11.3	10.7	7.8
	1000	12.1	11.3	6.3	10.9	10.5	7.8

*This was compared with the effect of these three-way interactions on the relative expression of both a structural gene and a regulatory gene (aflD, aflR) in modified a_w_ × temperature × 650 and 1000 ppm CO_2_*.

Table 3 summarizes the results of this study and shows the effects of the three-way interacting conditions on relative gene expression (*aflD*, *aflR*) and AFB_1_ production. This clearly shows that under slightly elevated CO_2_ conditions there was a stimulation of AFB_1_ production, especially under drought stress at 37°C and 650 and 1000 ppm CO_2_ exposure. It seems that the interactions between these three factors together are critical in the impact that slightly elevated CO_2_ has. This is clear from the results obtained at 0.92 and 0.95 a_w_ × 37°C and 650 or 1000 ppm CO_2_ where a statistically significant increase in AFB_1_ was observed.

**Table 3:** **Summary of the impact that interactions between the three climate change variables have on relative expression of the structural and regulatory genes (*aflD*, *aflR*), and aflatoxin B_1_ production (from Medina et al., in press)**.

**Temperature (°C)**	**a_w_**	**CO_2_ (ppm)**	***aflD***	***aflR***	**AFB_1_**
34	0.97	650	=	=	=
		1000	=	=	=
	0.95	650	=	=	=
		1000	=	↑(×3.6)	=
	0.92	650	=	↑↑(×24.4)	↑(×2.6)
		1000	=	↑(×2.0)	↑(×2.0)
37	0.97	650	↑(×4.6)	=	↑↑(×30.7)
		1000	↑(×6.5)	=	↑↑(×23.8)
	0.95	650	↑(×6.4)	↑↑(×14.6)	↑↑↑(×79.2)
		1000	↑(×3.2)	↑↑(×43.9)	↑↑↑(×78.5)
	0.92	650	=	↑↑(×40.4)	↑↑(×15.1)
		1000	↑↑(×22.5)	↑↑↑(×1680)	↑↑(×23.8)

*=, variation lower than 2-fold. Numbers between brackets refer to the fold-variation with respect to the control*.

## Discussion and conclusions

This review has considered the impact of different key environmental factors on the growth, gene expression and AFB_1_ production by *A. flavus*. This has shown that while there are some examples of the impact that changes in climatic weather conditions may have resulted in a switch with contamination from fumonisins to aflatoxins in maize, there have been few studies to examine the three-way interactions of the key environmental factors. Previous studies have examined water stress × temperature interactions on relative biosynthetic genes involved in aflatoxin production and that by other mycotoxigenic fungi (Abdel-Hadi et al., 2010; Schmidt-Heydt et al., 2010; Medina et al., 2013). However, there has been little detailed evidence of the impact that three-way interacting factors may impact on aflatoxin production.

The recent study by Medina et al. (in press) is the first to attempt to quantify the effects of interacting factors of water stress × temperature × elevated CO_2_ on growth, biosynthetic gene expression and AFB_1_ production. This new study suggests that when the three climate change factors are interacting there are responses which are not obtained when examining a_w_ × temperature conditions only. Thus, while growth is relatively unaffected by the addition of 2× and 3× existing CO_2_ levels at 37°C under the different water stress treatments used, this is not the case with mycotoxin production. The relative increased expression of both the structural *aflD* and the regulatory *aflR* genes in this recent study suggests that there is a significant impact on the biosynthetic genes involved in secondary metabolite production by strains of *A. flavus*. This was especially so at 37°C and under water stress (0.95, 0.92 a_w_) where more changes were observed. This study showed that there is a strong stimulation of mycotoxin production (from ×15.1 to ×79.2 depending on the climate exposure conditions used).

Additional studies are now required to evaluate whether this is a general stress response or whether the presence of elevated CO_2_ results in its incorporation into the biosynthetic pathways for enzyme production and secondary metabolite production. Perhaps new studies need to be carried out with the cell wall integrity (CWI) and high-osmolarity glycerol (HOG) pathways to examine whether they are triggered by stimuli of the three interacting factors of water stress × temperature × elevated CO_2_ or if this is a general stress response *per se* (Hayes et al., 2014). Work is in progress with maize grain to compliment the data obtained by Medina et al. (in press). A recent study by Vaughan et al. (2014) showed that twice the existing CO_2_ concentrations (400 and 800 μmol CO_2_mol^−1^) increased the susceptibility of maize to *Fusarium verticillioides* proliferation although fumonisin B1 mycotoxin production was not affected. They showed that inoculation at silking the accumulation of sugars, free fatty acids, lipoxygenase transcripts, phytohormones, and downstream phytoalexins were reduced in the maize grown at elevated CO_2_ conditions. Further studies using this approach are required where maize is grown under such conditions and then examining the host-pathogen interaction under the climate change scenarios described here.

Abdel-Hadi et al. (2012) with the aim of forecasting the AFB_1_ production by *Aspergillus flavus* examined the integration of growth, gene expression of multiple aflatoxin genes and AFB_1_ production by using a mixed secondary metabolite model. This model was validated at 37 and 40°C and different water stress levels and predicted AFB_1_ production at 37°C under water stress conditions, but none at 40°C. However, CO_2_ was not included in this model. The results obtained suggest that this model could be extended to include CO_2_ as a parameter and that this could be a very interesting tool to help in predicting the impact of climate change scenarios with experimental data sets as opposed to being based on historical data sets. This would be beneficial in quantifying impacts of climate change scenarios on economically important staple food crops.

There are some examples of previous studies using data on drought stress × temperature effects on *A. flavus* to predict impacts of interacting environmental factors. Work by Chauhan et al. (2008, 2010) demonstrated that it is possible to utilize an Agricultural Production Systems Simulator to calculate an Aflatoxin Risk Index (ARI) in both maize and peanuts in Australia. For maize they related seasonal temperature and soil moisture during the critical silking period to determine the ARI. They showed that both dry and hot climates made maize prone to a much higher aflatoxin contamination risk. For peanuts, they used the fractional amounts of available soil water during the crucial pod-filling period to determine the ARI. This showed that historically there has been an increase in aflatoxin contamination of peanuts in Australia related to increases in ambient temperature and decreases in rainfall. This has been developed into a web-interface tool for practically real-time use of this model. This approach is very valuable to predict low and high risk years in relation to climatic fluxes and may have application in West Africa where maize is also an important staple crop. However, these models may need modification to provide accurate predictions under climate change scenarios. Recently, Battilani et al. (2013) developed a mechanistic weather-driven model based on the infection cycle of *A. flavus* on maize to predict the risk of aflatoxin contamination in field on a daily basis from silk emergence to harvest. This included a probability index to exceed the legal limit of 5 μg/kg maize for aflatoxin. They suggested that this approach can be used for prediction of *A. flavus* infection and aflatoxin contamination during the growing season and at harvest. It may be possible to input the type of data from the present study to make this approach more accurate and improve the predictions of relative risk to take account of climate changes.

Many of the recent reviews which have examined aspects of the impact of climate change have focused on plant breeding, plant diseases and mycotoxins in Europe, Australia, Africa, and the USA (Boken et al., 2008; Chauhan et al., 2008, 2010; Wu et al., 2011). These have predominantly examined the existing or historical information and tools relevant to the impacts on crop yield, the impact of drought episodes and lack of water or elevated temperatures. Magan et al. (2011) examined the impacts of a_w_ × temperature stress on potential changes in mycotoxin production when the temperature is changed by +3 and +5°C and under different water stress regimes. Other reviews have used this same data (Paterson and Lima, 2010, 2011, 2012) to make their prediction of potential impacts. However, these previous studies did not include the three way interactions between a_w_ × temperature × elevated CO_2_. The laboratory based studies now available and those being done in FACE based systems need to be combined to be able to obtain more accurate information which can be used to predict on a regional basis the real impact that climate change scenarios may have on exposure to aflatoxins. This is especially important in at risk regions such as parts of Africa and Asia where the risks of exposure may increase under these predicted climate change conditions and threaten food security.

### Conflict of interest statement

The authors declare that the research was conducted in the absence of any commercial or financial relationships that could be construed as a potential conflict of interest.
